# Design and testing of microbubble‐based MRI contrast agents for gastric pressure measurement

**DOI:** 10.1002/mrm.27992

**Published:** 2019-09-16

**Authors:** Edwin Abdurakman, Martin Bencsik, Gareth W. V. Cave, Caroline L. Hoad, Scott McGowan, David J. Fairhurst, Giles Major, Penny A. Gowland, Richard Bowtell

**Affiliations:** ^1^ Department of Physics & Mathematics School of Science & Technology Nottingham Trent University Nottingham United Kingdom; ^2^ Division of Radiography & Midwifery School of Health Sciences, City University of London United Kingdom; ^3^ Department of Chemistry & Forensic School of Science & Technology Nottingham Trent University Nottingham United Kingdom; ^4^ Sir Peter Mansfield Imaging Centre School of Physics & Astronomy University of Nottingham Nottingham United Kingdom; ^5^ National Institute for Health Research (NIHR) Biomedical Research Centre Nottingham University Hospitals NHS Trust and University of Nottingham Nottingham United Kingdom; ^6^ Nottingham Digestive Diseases Centre, School of Medicine University of Nottingham Nottingham United Kingdom

**Keywords:** alginate, functional dyspepsia, microbubbles, MRI contrast agents, stomach pressure

## Abstract

**Purpose:**

This work demonstrates specifically tailored microbubble‐based preparations and their suitability as MRI contrast agents for ingestion and measuring temporal and spatial pressure variation in the human stomach.

**Methods:**

Enhanced alginate spheres were prepared by incorporating gas‐filled microbubbles into sodium alginate solution followed by the polymerization of the mixture in an aqueous calcium lactate solution. The microbubbles were prepared with a phospholipid shell and perfluorocarbon gas filling, using a mechanical cavitational agitation regime. The NMR signal changes to externally applied pressure and coming from the enhanced alginate spheres were acquired and compared with that of alginate spheres without microbubbles. In vivo investigations were also carried out on healthy volunteers to measure the pressure variation in the stomach.

**Results:**

The MR signal changes in the contrast agent exhibits a linear sensitivity of approximately 40% per bar, as opposed to no measurable signal change seen in the control gas‐free spheres. This novel contrast agent also demonstrates an excellent stability in simulated gastric conditions, including at body temperature. In vivo studies showed that the signal change exhibited in the meal within the antrum region is between 5% and 10%, but appears to come from both pressure changes and partial volume artifacts.

**Conclusion:**

This study demonstrates that alginate spheres with microbubbles can be used as an MRI contrast agent to measure pressure changes. The peristaltic movement within the stomach is seen to substantially alter the overall signal intensity of the contrast agent meal. Future work must focus on improving the contrast agent's sensitivity to pressure changes.

## 1 INTRODUCTION

Several studies^1^, ^2^, ^3^ have shown that suitably prepared gas‐filled microbubbles are useful as a pressure probe for MRI. These microbubbles create spatial magnetic field perturbations in their surrounding liquid medium and thereby alter the measured MR signal. Additionally, the flexibility of these biocompatible bubbles enables them to change in size due to a change in pressure, resulting in a measurable MR signal change.^4^ In our previous work, we showed the microbubble's ability to provide the user with a measurement of a spatial pressure gradient by means of MRI.^5^ Currently, this type of contrast agent is only commercially available as an injectable fluid solution for ultrasound imaging purposes,^6^ and to date, no clinical application has been shown for MRI.

A clinical condition known as functional dyspepsia is thought to arise from disruption of the normal function of the stomach, such that a failure of gastric accommodation and relaxation leads to abnormal variations in the pressure exerted by the stomach on the meal.^7^ Two clinical currently available methods, manometry^8^ and barostat,^9^ have shown promise in assessing pressure in the human stomach, but both of these techniques are invasive and can perturb the true physiology of the stomach.

Symptoms of functional dyspepsia have been shown to intensify immediately after ingesting a meal.^9^ In this study, to further understand this disease, we suggest a noninvasive method to simulate a meal and simultaneously measure the dynamic pressure distribution in the stomach. Use of a fluid meal to map pressure changes in the stomach's different areas would be challenging because pressure will instantaneously equilibrate throughout the meal. This issue can be overcome by using a soft‐solid edible substance, such as a large collection of small alginate spheres, as an artificial meal, allowing measurements of pressure changes due to local compression.

Alginates are polysaccharide compounds that are extracted from brown seaweeds,^10^ where their main role is to provide a structural matrix to support a cell. In various applications,^11^ alginates are commonly reacted with calcium ions to create a more elastic and stable gel,^12^ which is used widely in the food industry as a thickening and stabilizing agent.^13^ There have been studies investigating the behavior of alginate spheres in gastro‐intestinal conditions,^14^ but not in the context of MRI pressure probing.

In this study, we developed a pressure‐sensitive MRI contrast agent by entrapping microbubbles in alginate gel spheres and assessed its MR sensitivity to measure pressure changes at body temperature, and in a simulated gastric acid solution. Magnetic resonance imaging in vivo pilot studies were also undertaken in which healthy volunteers ingested the contrast agent meal immediately before the MRI procedure.

## 2 METHODS

### 2.1 Preparation of microbubbles

Microbubbles with diameters in the range of 2‐3 μm^1^ were prepared with a 1,2‐dihexadecanoyl‐sn‐glycero‐phosphocoline (DPPC) and 1,2‐distearoyl‐sn‐glycero‐3‐phosphoethanolamine‐*N*‐[methoxy (polyethylene glycol) ‐ 2000] (DSPE‐mPEG2000) (Avanti Polar Lipids, Alabaster, AL) as lipids shell and filled with octafluoropropane (C_3_F_8_) gas (Alchimia, Italy). The microbubble solution was prepared and analyzed following the procedure explained by Bencsik et al.^1^

### 2.2 Preparation of enhanced alginate spheres

The sodium alginate solution (2% weight/volume) was prepared by gradually adding a sodium alginate powder (Special Ingredients, Chesterfield, United Kingdom) into distilled water at room temperature and stirred constantly for 2 hours. This solution was left to degas spontaneously for 4 hours and was then placed in a high vacuum pump to further degas it. To produce the enhanced alginate spheres, the microbubbles that were prepared from the previous step were slowly introduced into the gel solution (20 mL) and then dripped into a calcium lactate solution (0.5% weight/volume) where they were left to cure for 10 minutes. In a separate study also presented in this paper, half of the volume of sodium alginate gel (10 mL) was used to make alginate spheres containing twice the bubble density of the previous preparation.

### 2.3 Preparation of the simulated gastric acid solution

A simulated gastric solution was prepared following the formulation shown in a previous study.^15^ A mixture of sodium chloride (2.86 g), potassium chloride (0.865 g), and calcium chloride (0.4 g) were diluted into 1 L of water. Then, while constantly stirring with a magnetic stirrer, hydrochloric acid was added gradually into the solution to reduce the pH to 2.0.

### 2.4 Magnetic resonance imaging experiment I: in vitro studies

The MRI experiments in the following sections were carried out at 2.35 T on a Bruker BIOSPEC (Billerica, MA) at Nottingham Trent University, or at 3 T using a whole‐body Philips Achieva scanner (Amsterdam, Netherlands) in the Sir Peter Mansfield Imaging Centre, University of Nottingham, United Kingdom.

#### 2.4.1 Mapping effective transverse relaxation time, $T_{2}^{\mathrm{eff}}$, with pressure changes

A single‐compartment sample holder was filled with enhanced alginate spheres in water and then further connected to a pump and a pressure gauge. In this experiment, quantitative $T_{2}^{\text{eff}}$ images were made using the multislice multi‐echo sequence^16^ every 9 minutes, and the pressure was very slowly increased, from 0 to 1 bar, within 2.5 hours, allowing 15 separate high‐resolution $T_{2}^{\text{eff}}$ maps to be made.

#### 2.4.2 Effect of microbubbles on the $T_{2}^{\mathrm{eff}}$

**Figure 1:**
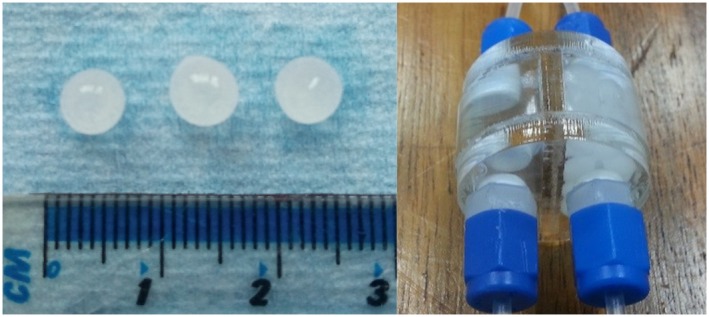
Left: Alginate spheres with microbubbles appear milky and the size is approximately 5 mm. Right: Twin‐compartment sample holder containing both preparations of alginate spheres in water suspension, equipped with Swagelok (Solon, OH) connectors, enabling the pressure being applied into the system to be changed

In this experiment, 2 preparations of alginate spheres were made, 1 without and 1 with entrapped microbubbles (Figure 1). The spheres were loaded into a twin‐compartment sample holder to facilitate the simultaneous MR assessment of both preparations, and then further connected to a single pressure gauge and pump, allowing the pressure to be changed and monitored during MRI scanning.

Before the application of any pressure, the sample was imaged in its 2‐compartment container using a multislice multi‐echo sequence to measure the spatial distribution of $T_{2}^{\text{eff}}$ in both preparations. This also allows further optimization of the next MRI sequence and provides evidence on the bubble's spatial distribution within the spheres.

#### 2.4.3 Nuclear magnetic resonance signal changes to rapid pressure cycling

Pressure changes were simultaneously applied to both compartments. At the same time, RARE ($T_{2}^{*}$‐weighted) images were acquired with pressure cycling between 0 and 1 bar, 5‐mm thickness at TE of 10 ms every 7 seconds (= TR), and the effective TE (TE^eff^) set to 400 ms, dictated by the $T_{2}^{\text{eff}}$ assessment undertaken in the previous experiment.^1^

#### 2.4.4 Magnetic resonance imaging sensitivity with different bubble densities, and effect of an acid simulating gastric conditions

Two preparations of alginate spheres were used with different bubble densities, one identical to that used previously (4.5% volume fraction), and another one with approximately twice more bubbles, to demonstrate the resulting increase in MR sensitivity to pressure change. The 2 different alginate spheres preparations were loaded into the separate compartments of the sample holder.

Next, RARE images were acquired with pressure cycling between 0 and 1 bar, using the same sequence parameters as described before, initially in the presence of water. Then, the pressure cycling was stopped, and the water surrounding the alginate spheres was flushed out and replaced with the solution simulating gastric acid, before the pressure cycling and MRI were resumed.

#### 2.4.5 Magnetic resonance imaging sensitivity at body temperature

Alginate spheres with microbubbles were placed in a single‐cell sample holder in water at room temperature and then further connected to a pump and a pressure gauge. The RARE images were acquired with pressure cycling between 0 and 1 bar, using the same sequence parameters as described previously.

First, the pressure was cycled for approximately 12 minutes between 0 and 1 bar. The pressure cycling was then stopped, and the water surrounding the alginate spheres was flushed out and replaced with water at 37°C.

#### 2.4.6 Demonstrating a spatial gradient

In this experiment, the alginate spheres were sieved out of the water suspension and then placed in an empty plastic bottle (50 mL). Additionally, a deflated balloon was connected to a bicycle air pump, enabling its inflation, and then placed in the plastic bottle containing the alginate spheres. This way, after inflation, the balloon can compress the spheres as it changes size, at varying locations within the vessel, allowing different alginate spheres to experience different pressures in a way similar to what we hope to see in the case of them residing in the human stomach.

The sample was imaged using a RARE sequence in a sagittal plane with 9 contiguous, 4‐mm‐thick slices. We monitored the MR signal changes coming from the spheres for 23 minutes as the balloon pressure was cycled between 0 and 1 bar, expanding its outer shape to different sizes. The pressure was made to reach 1 bar very quickly, and it was then slowly released in steps of 0.1 bar.

#### 2.4.7 Magnetic resonance imaging sensitivity in locust bean gum and acid solution simulating gastric conditions

Because of the lowered mass density of alginate spheres with microbubbles, they naturally float when placed in a water suspension. Thus, a viscous locust bean gum (2% weight/volume) has been used as a suspending medium to help sustain their homogenous distribution across the entire volume of the meal. A total of 250 mL of alginate spheres with microbubbles was mixed with 250 mL of locust bean gum solution in a plastic bottle at room temperature, simulating the actual volume to be ingested by a volunteer in our in vivo studies.

An inlet was created, enabling the bottle to be connected to a pump and allowing the pressure to be changed during the MRI scanning. An hour before the experiment, the sample was placed in a water bath (37°C), followed by 50 mL of the sample being scooped out and replaced with the same volume of acid simulating gastric solution at 37°C. Immediately after the pump was switched on, MR images were acquired using the RARE sequence (TE^eff^ = 500 ms) with 3 slices at 5‐mm thickness, every 7 seconds. The pressure was continuously cycled between 0 and 1 bar until the MRI scanner sequence was finished within a 10‐minute‐long experiment.

#### 2.4.8 Exploring the effect of the meal heterogeneity

We anticipate that it is possible for the meal MR image heterogeneity to contribute to the alteration of the region of interest (ROI) signal intensity from shot to shot within the dynamic images when it resides in the stomach. To investigate this effect, we performed an experiment to quantitate the signal intensity change as the slice selection is intentionally moved throughout the sample, on which no pressure was applied during the MRI acquisition. In this way we assessed the signal intensity changes due to the meal heterogeneity (or other causes, excluding that of pressure variations).

Alginate spheres with microbubbles were prepared and suspended in distilled water, then loaded into a Perspex container (100 mL) before being placed in a water bath at 37°C. The sample was placed in the scanner in a vertical orientation. The MR images were obtained using a fast imaging sequence, the balanced turbo field echo (BTFE)^17^, ^18^ (T_2_‐preparation time = 100 ms, TE = 1.7 ms, TR = 3.4 ms), by changing the slice location along a vertical direction starting from the top toward the bottom of the sample, without applying any pressure. The data set was obtained with a slice thickness of 5 mm, and the slice selection was moved in 5‐mm increments across the sample, resulting in 8 slices.

### 2.5 Magnetic resonance imaging experiment II: in vivo studies

For in vivo studies, the contrast agent was prepared using food‐grade ingredients in the form of a meal to be consumed by the volunteers before being further scanned with MRI. An initial scan was performed using the RARE sequence, which resulted in severe motion artifacts. We therefore further explored the BTFE sequence to minimize these.

#### 2.5.1 Selection of participants

The ethical approval for the in vivo studies was obtained from the ethics committee of the University of Nottingham (Approval number B18022016). Participation into the study was entirely voluntary, and all the volunteers gave written informed consent. Four volunteers (2 males, 2 females, age range 25‐55 years) were recruited for 5 studies. All volunteers had no history of gastrointestinal disease and were suitable for MRI scanning. The first 4 studies were used to gradually improve our MRI protocol by (1) decreasing movement artifacts by changing the MRI sequence from RARE to BTFE, (2) assessing the signal changes with and without microbubbles, (3) assessing the partial volume effect minimization obtained by using respiratory triggering, and (4) optimizing the BTFE T_2_‐preparation time. Only the fifth study provided results showcased in our main manuscript.

#### 2.5.2 Meal ingestion protocol

The contrast agent meal (500 mL), warmed up to 37°C, was placed into 2 separate plastic cups. Before consuming it, participants were asked to ingest a high‐fat content meal, 50 mL of Calogen (Nutricia, Trowbridge, United Kingdom), to rapidly turn the stomach from a fasted into a fed state and to help retain the alginate meal in the stomach for a longer time duration.^14^ After 15 minutes, the participants were asked to eat as much as they felt comfortable of the contrast agent. The volunteers were encouraged to ingest the spheres without chewing to retain their shape and functionality in sensing pressure in the stomach. They then underwent an approximate 30‐minute‐long MRI scan.

#### 2.5.3 Magnetic resonance imaging in vivo investigations with respiratory triggering

Before this study, scanning was performed on 1 volunteer using the RARE sequence, which resulted in severe motion artifacts. During normal breathing, the stomach moves and causes a different location of the tissue to be captured in each of the MR images at a specific location, which for this study also contributes to the ROI signal variations in the meal, hindering the accurate quantitation of the signal that solely results from the pressure change in the stomach. Our strategy to overcome this issue was to use a fast imaging sequence (BTFE) as well as using respiratory triggering. This way, the acquired dynamic images were expected to be more steady from one image to another. First, the experiments were performed on the alginate spheres without microbubbles to establish any MR signal changes excluding the effect of pressure changes. Then, a further investigation was carried out on the meal with the presence of the microbubbles.

#### 2.5.4 Magnetic resonance imaging in vivo image analysis

**Figure 2:**
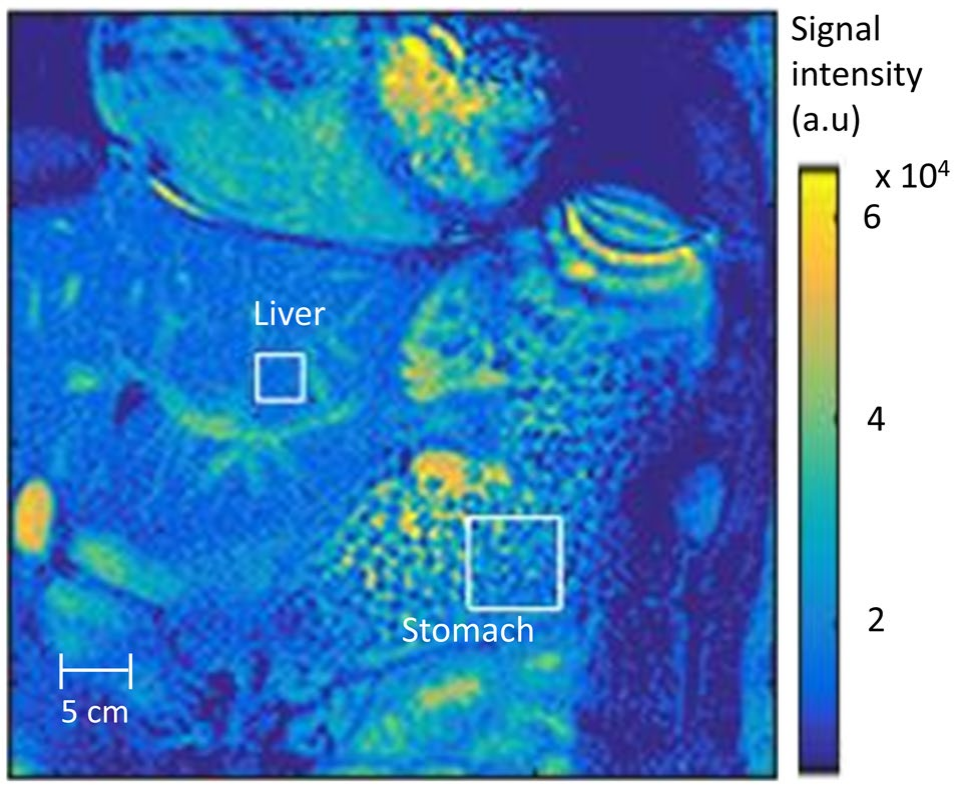
Magnetic resonance image showing the meal alginate spheres with microbubbles in the stomach with the surrounding organs, acquired with the balanced turbo field echo sequence with the respiratory gating. The white squares highlight the region of interest selections for the signal intensity ($T_{2}^{*}$‐weighted) analysis in the meal and the liver

For the image analysis, the software was written to allow a specific ROI tracking from frame to frame, using a cross‐correlation product analysis with the interpolated image of the first frame on the selected ROI. A square‐shaped ROI from the meal was selected, while another one from a small section of the liver (Figure 2) served as a reference, because signal intensity within this region is expected to have less deviation. The ROI tracking was undertaken to try to have the selected ROIs within the exact same tissue area in each of the acquired dynamic images, in spite of small lateral shifts taking place in the raw images. The tracking inherently fails in a landmark‐free homogeneous area; hence, the successful tracking of the ROI in the liver required the selection of a second ROI with heterogeneous structure, including clearly distinct blood vessels within the liver. Then, after successful tracking of the corresponding tissue, plots of the signal intensity coming from both of these areas are produced.

## 3 RESULTS

### 3.1 Effective transverse relaxation time, $T_{2}^{\mathrm{eff}}$, with pressure

**Figure 3:**
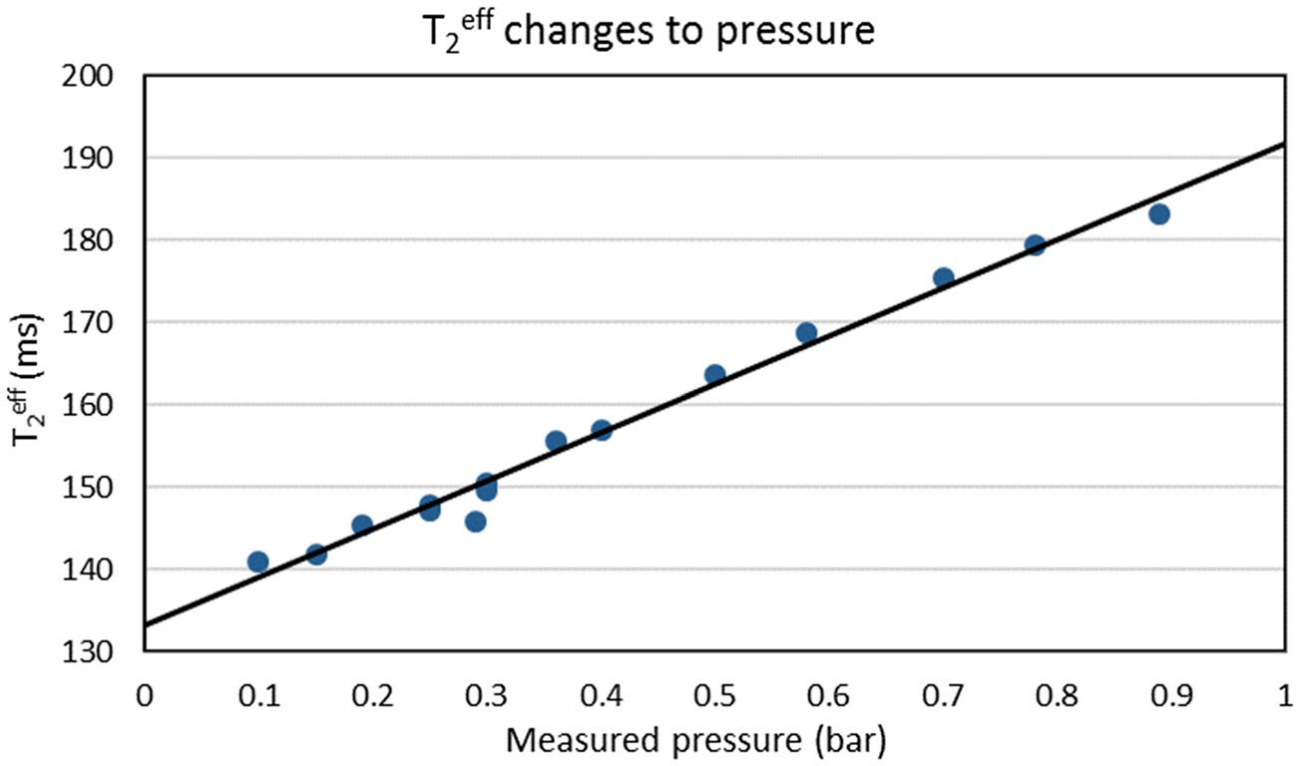
Graph showing a linear correlation between the effective transverse relaxation time, $T_{2}^{\text{eff}}$, of microbubble‐loaded alginate spheres with pressure change. The $T_{2}^{\text{eff}}$ increases as the pressure being applied increases, with a sensitivity of 43% change per bar

We estimated the mean $T_{2}^{\text{eff}}$ value of the sample using custom‐written MATLAB (MathWorks, Natick MA) code by selecting the pixels corresponding to alginate spheres with microbubbles for each applied pressure. As a result, a linear correlation is demonstrated with a clear increase of $T_{2}^{\text{eff}}$ with increasing measured pressure seen all over the spheres, with a sensitivity of 43% $T_{2}^{\text{eff}}$ change per bar (Figure 3). Although this shows that, as expected, our sensitivity comes from the susceptibility effect, the direct measurement of the MR transverse relaxation is usually difficult and time‐consuming; therefore, further assessments of signal changes in our work are all done entirely by the acquisition of rapid $T_{2}^{*}$‐weighted images.

### 3.2 Effect of the presence of microbubbles on $T_{2}^{\mathrm{eff}}$

**Figure 4:**
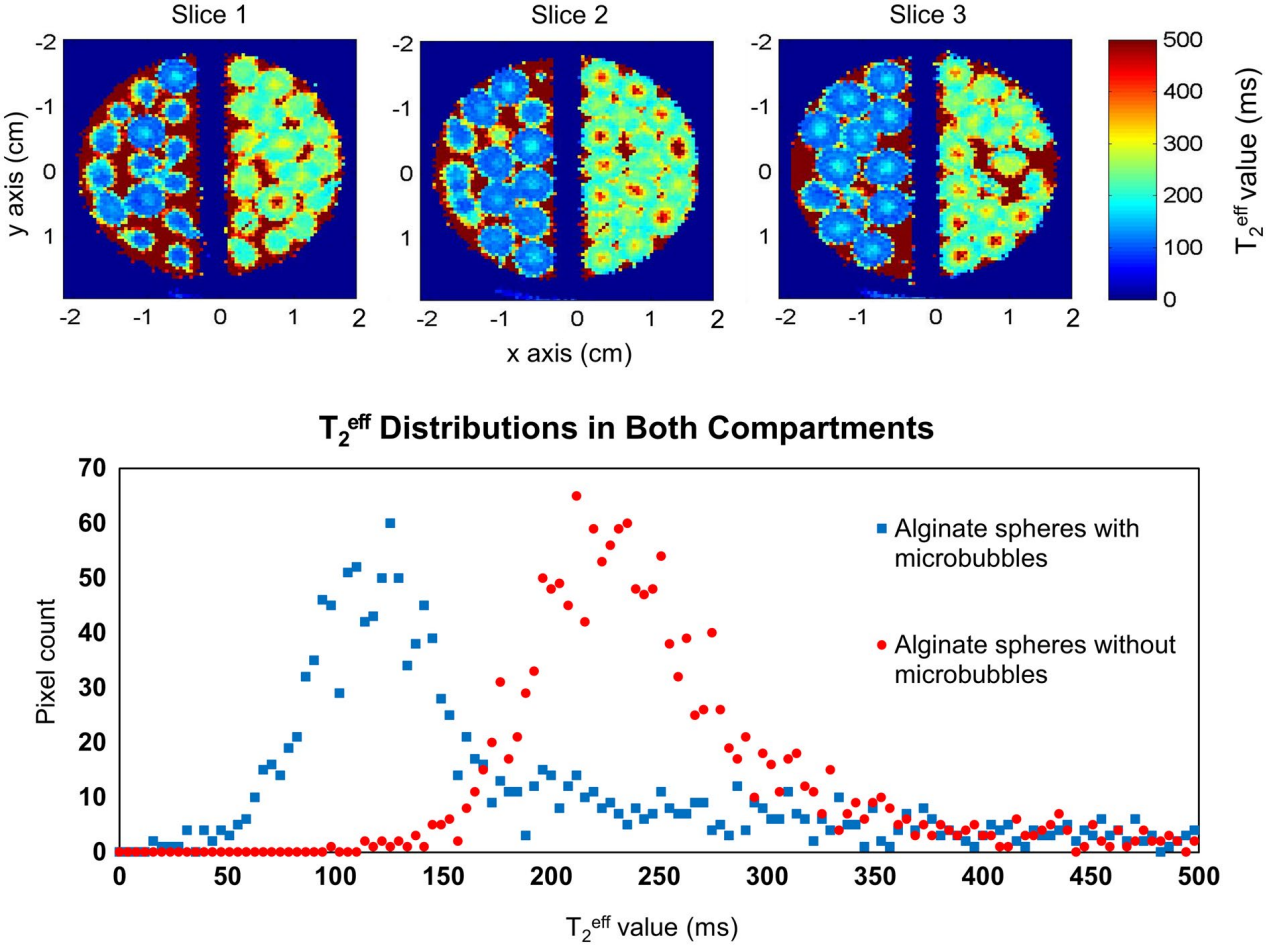
Top: Three consecutive MR images showing $T_{2}^{\text{eff}}$‐weighted alginate spheres with (left compartment) and without (right compartment) microbubbles. Bottom: Plot showing the pixel intensities distribution extracted from specific regions of interest coming from the images above, demonstrating the $T_{2}^{\text{eff}}$ difference between both preparations. The $T_{2}^{\text{eff}}$ value is substantially lower in the alginate sphere with microbubbles and toward the periphery of the spheres

Figure 4 shows 2 clearly differentiated compartments, demonstrating that $T_{2}^{\text{eff}}$ is substantially reduced in the presence of microbubbles, approximately by a factor of 2. The $T_{2}^{\text{eff}}$ values are about 200 ms to 300 ms for the spheres without microbubbles and between 100 ms and 200 ms for the spheres with microbubbles. Additionally, there is evidence of a radial spatial gradient of $T_{2}^{\text{eff}}$ value between the core and the periphery of alginate spheres with microbubbles. The microbubbles density and the curing process is likely to differ spatially between these 2 areas.

### 3.3 Magnetic resonance imaging sensitivity of alginate spheres to rapid pressure cycling

**Figure 5:**
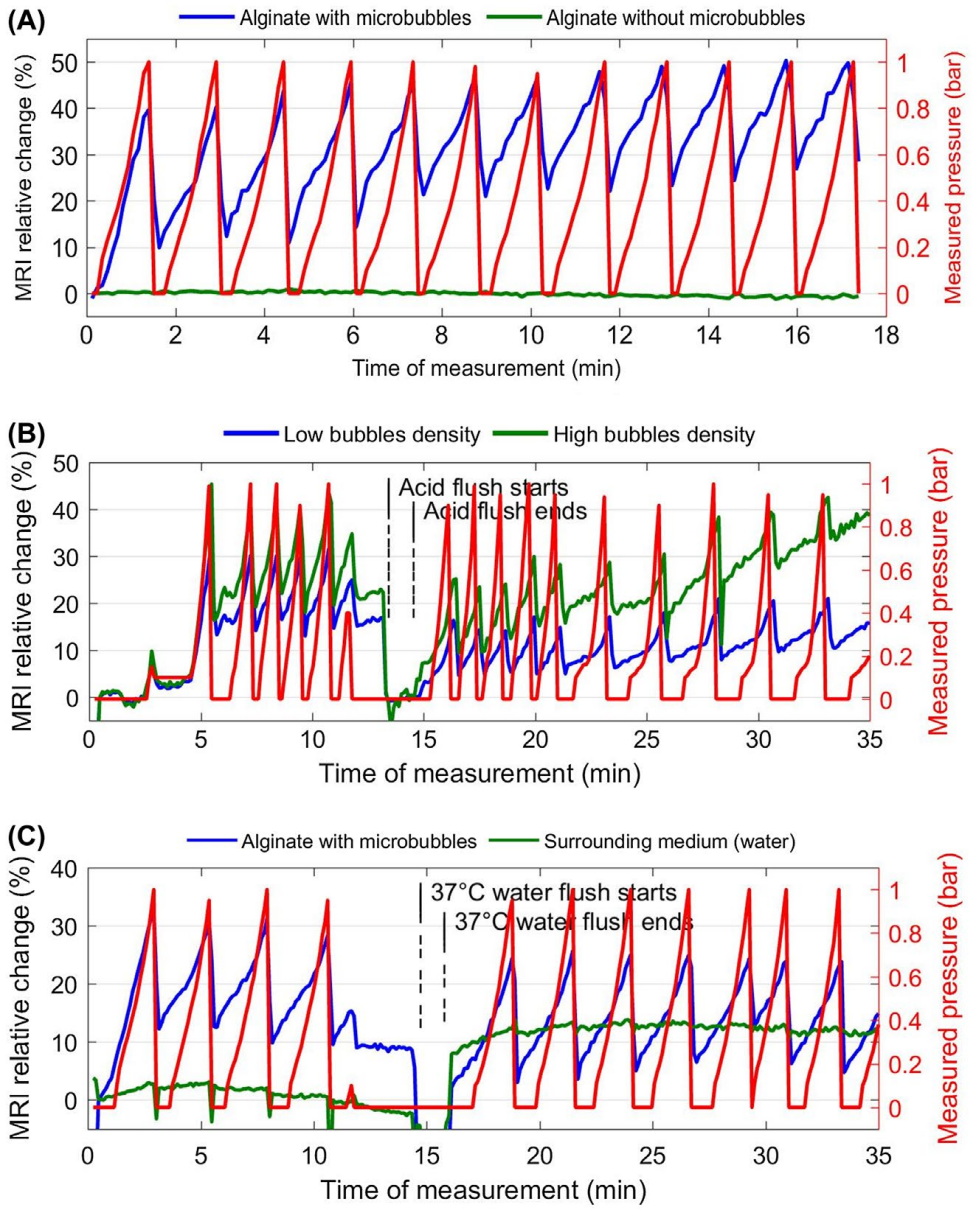
A, Relative signal intensity ($T_{2}^{*}$‐weighted) changes in 2 compartments: alginate spheres with bubbles typically showing 40% signal change per bar, while the signal intensity (SI) remains constant in alginate without bubbles. The pixels were selected so as to eliminate the water solvent from the analysis, to ensure that the SI comes solely comes from the spheres. The red curve shows the measured pressure. B, Comparison of the sensitivity to pressure changes between low and high microbubble densities: approximately 10% difference both in water and in solution, mimicking the gastric acid. The time measurement from 0 to 13.5 minutes corresponds to the relative signal change of alginate spheres in the presence of water, and 14 minutes onward in the acid. C, Sensitivity of the alginate spheres with microbubbles in a warm (body temperature) surrounding water remains the same as in room temperature (18°C). The measurement from 0 to 14 minutes corresponds to the relative signal change from alginate spheres in water at 18°C, and 15 minutes onward at 37°C. The corresponding videos of these data are supplied in Supporting Information Videos [Link], [Link], [Link]

In the $T_{2}^{*}$‐weighted images, the MR signal intensity in the spheres with entrapped microbubbles exhibits changes of approximately 40% change per bar. On the contrary, the signal remains constant (±1% error) throughout the experiment in the spheres without microbubbles, demonstrating that the signal change is solely due to the presence of the microbubbles. There is a 10% signal drift seen over the entire time duration of the experiment, most probably due to bubble destruction; however, the sensitivity remains as high as 28% change per bar toward the end of this 17‐minute‐long experiment (Figure 5A) (Supporting Video S1).

In the experiment comparing 2 different bubble densities, in the initial pressure cycle in the presence of water, the sensitivity of alginate spheres with twice‐higher bubbles density is approximately 45% change per bar, and 35% in the lower density preparation (Figure 5B) (Supporting Video S2). This difference is less than expected, probably because at high enough density, the field gradients of individual bubbles will start overlapping, and the increase in MR sensitivity is not linear with bubble density anymore.^1^ Although there is a slight increase of MR sensitivity with bubble density, the result suggests that there is no great benefit in exploring even higher values of bubble density.

Following the introduction of simulated gastric acid, there is a 15% decrease in the overall $T_{2}^{*}$ of samples, as expected (Figure 5B). A previous study carried out by Rayment et al^15^ showed that, at a pH of 2.0, the value of T_2_ in alginate spheres considerably decreased, suggested to be caused by the shrinking and the formation of a denser acid gel network. The sensitivity of this contrast agent decreases by approximately 50% in the acid solution. However, the sensitivity difference between the 2 preparations, higher and lower bubble density, remains about 10% per bar as seen in water, but then slowly decreases to only 2% difference per bar after 15 minutes of the acid having been flushed. This could be due to the bubble destruction enhanced in a higher bubble density where there is a significant signal drift seen, approximately 17%. We demonstrated that, following the dipping of the alginate spheres in the acid, the contrast agent is still very much functional for at least 15 minutes.

In monitoring the sensitivity of this contrast agent at body temperature, when the warm water was introduced, the signal drops temporarily due to nuclear spins being displaced from the slice under investigation while the imaging takes place. Following the flushing, there is a slight decrease, approximately 8%, in the $T_{2}^{*}$ of the alginate spheres. However, the sensitivity of alginate spheres remains the same as seen at room temperature, about 35% per bar, for at least another 15 minutes (Figure 5C) (Supporting Video S3).

### 3.4 Magnetic resonance imaging sensitivity in the periphery and in the core of alginate spheres

**Figure 6:**
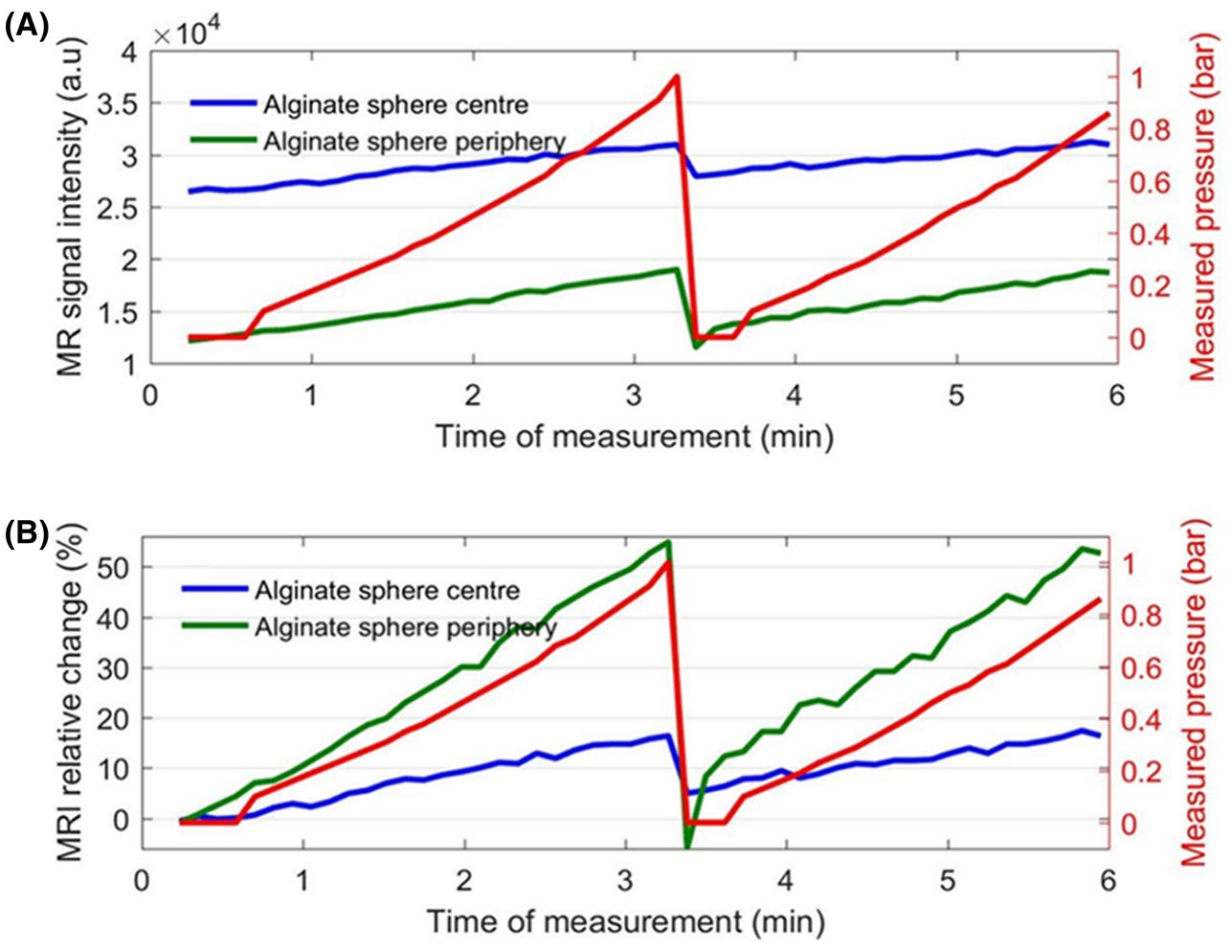
A, There is approximately 50% MR signal intensity ($T_{2}^{*}$‐weighted) difference between the core and the periphery of an alginate sphere, and it is lower in the periphery. B, The periphery exhibits higher sensitivity (55% change per bar) to pressure changes, compared with the core (18% change per bar). However, both regions contribute to the overall increase of the signal intensity in the alginate sphere. The red curves show the measured pressure

For this experiment, we analyzed the MR signal from a single pixel intensity coming from the center of a sphere and another single pixel intensity coming from the periphery of a sphere. The $T_{2}^{\text{eff}}$ value is higher, by a factor of 2, in the core compared with the periphery of the alginate sphere (Figure 4). We anticipated that the microbubbles density is higher in the periphery. This is consistent with the behavior of these spheres that we observed where the periphery exhibits higher sensitivity (55% change per bar) to pressure changes, compared with the core (18% change per bar) (Figure 6).

### 3.5 Spatial gradient effect

**Figure 7:**
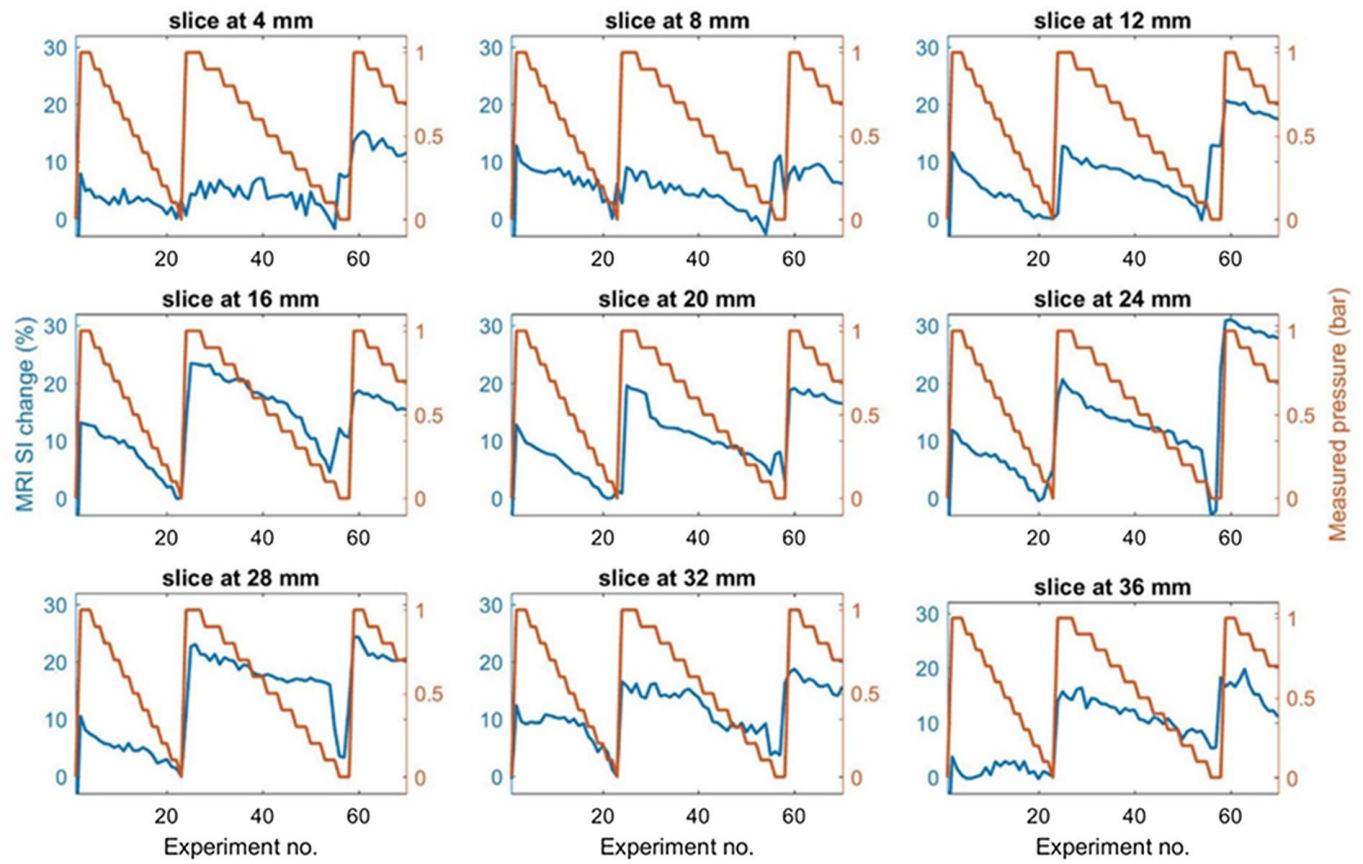
The graphs correspond to each slice of MR images throughout the sample, showing the mean SI exhibited ($T_{2}^{*}$‐weighted) on all 9 different image slices as the pressure is being cycled between 0 and 1 bar. The image analysis was undertaken in MATLAB by providing a threshold, and the highest SI change is seen on the image at slice position 24 mm and the lowest at slice 4 mm. This shows how the contrast agent is able to demonstrate pressure gradient on the sample. The blue and red curves represent the mean MR SI change and applied pressure, respectively

We analyzed the mean signal changes on each slice as the pressure is ramped up and down, and the result demonstrates differing signal changes at differing slices (Figure 7). The bubble‐enhanced alginate spheres located in different parts of the bottle exhibit differing signal changes, suggesting spatial gradients throughout the sample. The maximum signal change exhibited on the image at slice position 24 mm and the minimum at 4 mm, respectively, are 30% and 15% signal changes, as the pressure within the balloon gradually changed from 1 bar to 0 bar.

### 3.6 Magnetic resonance sensitivity in locust bean gum and simulated gastric acid solution

**Figure 8:**
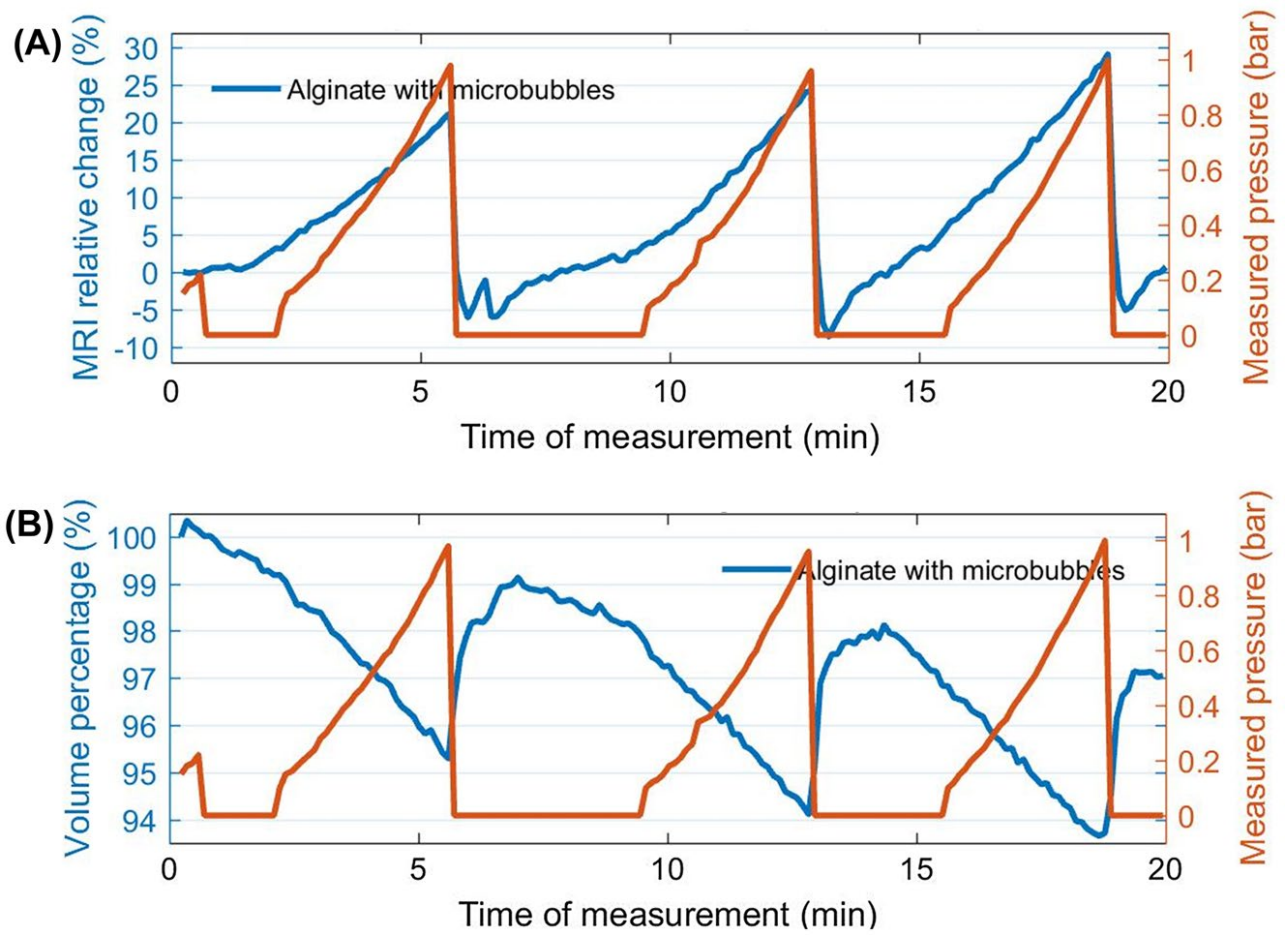
A, Graph showing that the sensitivity of microbubble‐loaded alginate spheres in locust bean gum with acid simulating gastric solution reaches approximately 22% signal change ($T_{2}^{*}$‐weighted) per bar. B, The alginate sphere volume decreases about 3% within this 20‐minute experiment due to exposure to acid. It also shows the volume change on the spheres due to the change in pressure. The red curves represent the measured pressure

The sensitivity of the contrast agent in a locust bean gum with simulated gastric solution is approximately 22% (Figure 8A). However, the sensitivity is seen to increase over time. The deterioration of the signal is best seen when observing the MR signal taking place at zero pressure. However, when applied pressure was released and the pressure gauge reading showed zero, sometimes a longer time was required for the pressure to come back to zero within the specimen. This could be due to the viscosity of the fluid in the thin tubing that links the pump to the sample holder. The maximum MR signal reached at the highest pressure, 1 bar, exhibits a remarkable steady state, perhaps due to the signal increase coming from bubble damage somewhat compensating for the signal decrease resulting from the acid. Overall, the locust bean gum is effective in immobilizing the spheres, while maintaining the robust signal sensitivity of this contrast agent.

Over time, the spheres are seen to shrink from the moment the acid solvent is added to the specimen. Thus, we estimated the volume change by using MATLAB processing to discriminate the volume of locust bean gum from that covered by the spheres, simply by setting a pixel intensity threshold. The results show that the acid‐driven volume shrinkage is approximately 3% within the 20 minutes of this experiment (Figure 8B).

### 3.7 Meal heterogeneity

**Figure 9:**
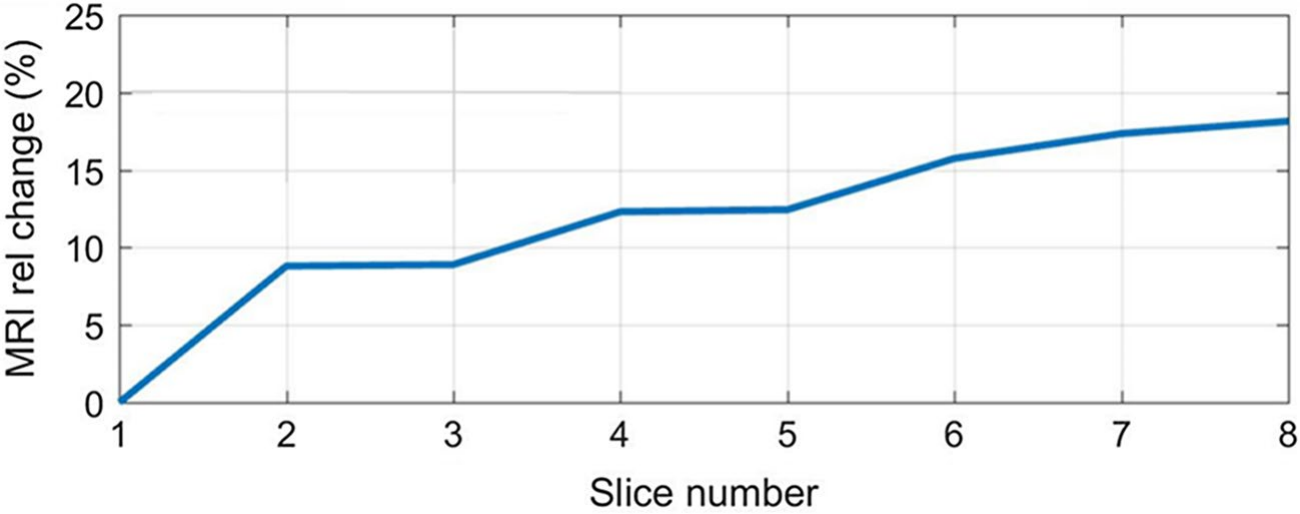
Plot showing the signal intensity ($T_{2}^{*}$‐weighted) change, up to 18% over a 10‐cm displacement, by shifting the position of the selected slice within the sample when there is no applied pressure. This demonstrates that the meal heterogeneity can contribute to the signal changes being captured when the slice selection moves relative to the meal

When the selected slice was moved throughout the sample, without applying any pressure, we calculated the relative signal change from 1 slice to another, using the first slice as the reference. As a result, the signal intensity varies substantially, up to 18% over a 10‐cm displacement (Figure 9). This particular result suggests that when the contrast agent resides in the stomach, it is likely that partial volume effect variations in the meal on different images will substantially contribute to the alteration of the signal intensity. This is one of the major limiting factors to be taken into consideration when the study proceeded to the in vivo experiment. This led us to systematically use respiratory‐triggered MR acquisition to minimize this undesirable artefact.

### 3.8 In vivo study: alginate spheres without microbubbles

**Figure 10:**
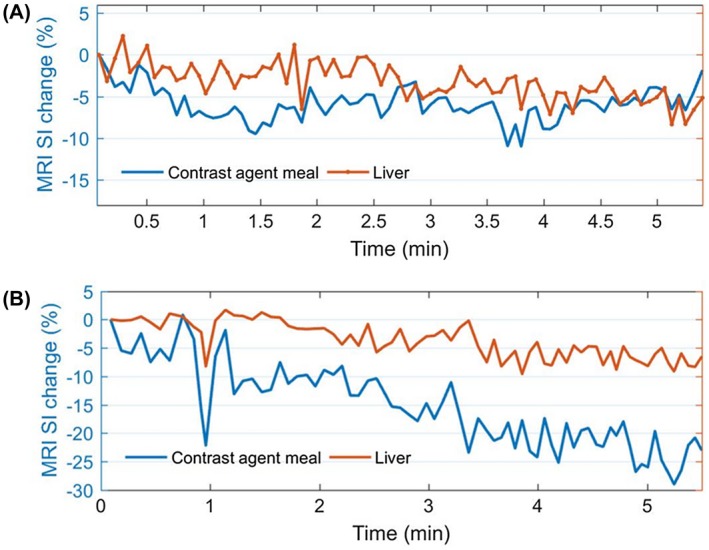
Signal intensity ($T_{2}^{*}$‐weighted) change in the meal: alginate spheres without microbubbles (A) and alginate spheres with microbubbles (B) and in the liver acquired with the balanced turbo field echo sequence with the respiratory triggering. The signal change in the meal of alginate spheres without microbubbles is lower (about 2% to 3%), compared to its counterpart meal with microbubbles (between 5% and 10%)

In the meal containing alginate spheres without microbubbles, the percentage of signal intensity change is about 2% to 3% with the respiratory gating (Figure 10A). However, there are also a few images exhibiting very large artifacts, coming from rare instances in which the scanner failed at tracking the volunteer's respiration. Moreover, large susceptibility artifacts appeared on all of the images caused by blood flow, empty space in the stomach, and lung displacement, which affect the signal substantially. The affected regions were avoided from the signal analysis and were not included in the signal deviations exhibited by the data shown on the graphs. A 5% signal drift can also be seen in the meal, which also appeared in the liver.

### 3.9 In vivo study: alginate spheres with microbubbles

The meal tracking, as previously explained, was also used for image analysis. However, it is much more robust here due to the severe heterogeneity of meal signal, thanks to the presence of the microbubbles. Initially, the signal intensity coming from the meal is seen to exhibit changes within 10%. However, the signal coming from the liver, which should be constant, is also seen to fluctuate within 5% (Figure 10). A similar effect has been exhibited previously with alginate spheres without microbubbles, and we suggest that this could be due to the effect of the T_1 _relaxation. Although there were certain instances in the measurement (from 1.1 minutes to 2 minutes) where the signal intensity in the liver is fairly constant (less than 1% changes), accompanied by 5% deviations in the stomach, the time duration of this occasion was too short to enable us to conclude that this was genuinely coming from pressure exerted by the stomach (Figure 10B).

In addition, similar to what can be seen in the alginate without bubbles, it appears that the overall signal intensities coming from both the meal and the liver are decreasing over time, with a signal drift of about 20% and 8%, respectively, in the meal and in the liver, within this 5.5‐minute observation. The enhanced drift seen in the meal is probably due to the effect of the acid on the meal.

## 4 DISCUSSION

In this work, we demonstrate that $T_{2}^{\text{eff}}$ relaxation time of alginate spheres is substantially reduced in the presence of gas‐filled microbubbles. For the experiments conducted where the pressure was gradually increased, $T_{2}^{\text{eff}}$ mapping results show an excellent linear correlation with the externally applied pressure. This is due to the change of the microbubble radius size in the alginate sphere as the pressure increases, causing the magnetic field perturbation to be altered, therefore changing the $T_{2}^{*}$ value.^4^

Our novel contrast agent exhibits very good stability, as it maintains its very high sensitivity throughout the rapid pressure cycle, including the case of simulated gastric conditions. The sensitivity usually demonstrated as high as 40% change per bar, due solely to the presence of microbubbles in alginate spheres. Although there is a slight sensitivity difference between the periphery and the core of alginate sphere, the overall $T_{2}^{\text{eff}}$ in the alginate sphere increases with the pressure. One possible explanation is that the bubble distribution is different between these 2 regions, because of the radially varying curing process of alginate in calcium solution.

The expected pressure exerted in the stomach on its meal, particularly in the antrum region, during the digestive period ranges between 30 mmHg and 82 mmHg (40 mbar to 109 mbar), with a frequency of about 3 waves per minute.^19^ Thus, considering the sensitivity of the contrast agent at 40% signal change per bar, the expected MR signal variation exhibited in the meal within the human stomach should range from 1.6% to 4.4%. Although we have not validated these figures experimentally, future work could use nasogastric intubation manometry to measure the pressure in vivo simultaneously with the MRI experiments.

In addition to its ability to demonstrate temporal pressure changes, this contrast agent allows us to probe a spatial pressure gradient by MRI. This is important because this promises the possibility of investigating the dynamic pressure distribution that takes place in a human stomach during meal processing.

When we increased the bubble density approximately by a factor of 2, the sensitivity increased approximately by 29%, which was much less than we expected. We suspect that at higher bubble density, the field gradients originating from individual bubbles start to overlap, causing the contrast agent's sensitivity to bubble density to leave linearity. Nonetheless, the sensitivity of the microbubbles to pressure variations can still be improved by exploiting the magnetic susceptibility of the microbubble shell and the mean bubble size.^20^ Although we have not explored this, the sensitivity can be further enhanced by incorporating magnetic materials on the shell of the bubble, such as iron nanoparticles.^21^

Unfortunately, in this study we also found that there is a signal intensity difference exhibited by shifting the location of the image slice selection within the sample. This is due to the MR signal heterogeneity within the meal, which can contribute to an unwanted signal change when it moves relative to the imaged volume. This effect is further evidenced and quantitated in the in vivo studies.

In the in vivo study on alginate spheres without microbubbles, the pressure change within the stomach should not bring any effect. However, the signal variations still measured in this meal with the use of respiratory triggering could be originating from T_1_ partial recovery, as the value of the TR cannot be set constant with the use of this technique. This could be affected by the change in respiration rate and depth of the participant over time. Additionally, a signal drift can be seen in the meal, which most probably results from the effect of the stomach acid. However, this also appeared in the liver to a lesser extent, which was not expected, and could be caused by the scanner electronic drift.

In the in vivo study on a meal containing alginate spheres with microbubbles, in spite of the respiratory triggering, some relative tissue displacements taking place from frame to frame can be seen as the meal is proactively moved by the stomach, and its elasticity makes the tracking of a portion of the meal very difficult. The meal pixel intensity is very heterogeneous locally within the length scale of the alginate spheres, due to the $T_{2}^{*}$ effect of microbubbles, and nonlocally on the length scale of the stomach, which is the brightest toward the antrum, as would be expected as the pressure there is highest. The meal exhibits little relative motion far away from the antrum, and far greater deviations from frame to frame in the antrum.

We cannot yet conclude that the signal intensity changes observed are solely due to the pressure changes, as the signal might also be affected by partial volume effects, inhomogeneous dilution of the meal, and movement through the imaging gradients. However, we can test this further in the future in 2 ways: (1) by increasing the MR sensitivity to pressure changes of the preparation, such as by exploring a different size of microbubbles or coating them with a shell to enhance the susceptibility step change, or (2) making the meal's MR signal more homogeneous, such as by matching the transverse relaxation of the locus bean gum medium to that of the alginate spheres.

## 5 CONCLUSIONS

We have effectively enhanced specific features of a microbubble‐based MRI contrast agent to suit the need for human ingestion, by incorporating them within soft‐solid alginate spheres. In this study, we have demonstrated that this preparation can be used as a pressure‐sensitive MRI contrast agent, exhibiting a sensitivity as high as 40% signal change per bar. We have also demonstrated its behavior in various conditions simulating the environment of the human stomach, suggesting that our preparation is a viable contrast agent for in vivo applications in assessing pressure changes. This contrast agent may be useful as a noninvasive technique for assessment of the pressure changes underlying meal‐related symptoms, particularly in patients with functional dyspepsia.

Magnetic resonance imaging in vivo studies involving healthy volunteers were undertaken to test the functionality of the contrast agent, to measure the dynamic pressure changes within the human stomach. Although the peristaltic movement within the stomach is seen to alter the overall signal intensity of the contrast agent meal, at this point we could not conclude that these signal intensity changes are due solely to pressure changes. The signal intensity change exhibited in the meal within the antrum region is between 5% and 10%. This is slightly higher than expected and should be around 1.6% to 4.3% when considering the typical 40% per bar sensitivity. The signal changes may result from (1) the T_1_ effect (MRI acquisition being triggered by respiratory gating); (2) tissue elasticity (slight ingress of varying tissues in different frames are seen in the analyzed ROIs); or (3) the SNR. We are presently looking into the effect of different microbubbles sizes in enhancing the contrast agents’ sensitivity to introduce a clinically relevant new tool.

## Supporting information


**VIDEO S1** Relative signal intensity ($T_{2}^{*}$‐weighted) changes in 2 compartments: alginate spheres with bubbles typically show 40% signal change per bar, whereas the SI remains constant in alginate without bubbles

**VIDEO S2** Comparison of the sensitivity to pressure changes between low and high microbubble densities: approximately 10% difference both in water and in solution mimicking the gastric acid. The time measurement from 0 to 13.5 minutes corresponds to the relative signal ($T_{2}^{*}$‐weighted) change of alginate spheres in the presence of water, and 14 minutes onward in the acid

**VIDEO S3** The sensitivity of the alginate spheres with microbubbles in a warm (body temperature) surrounding water remains the same as in room temperature (18°C). The measurement from 0 to 14 minutes corresponds to the relative signal ($T_{2}^{*}$‐weighted) change from alginate spheres in water at 18°C and 15 minutes onward at 37°C
